# Nasopharyngeal tuberculosis suspected of malignancy: A case report

**DOI:** 10.1097/MD.0000000000040920

**Published:** 2025-01-10

**Authors:** Da Hyun Chung, Gil Joon Lee

**Affiliations:** a Department of Otorhinolaryngology, Head and Neck Surgery, School of Medicine, Kyungpook National University, Kyungpook National University Chilgok Hospital, Daegu, South Korea.

**Keywords:** lymph node, lymphoma, nasopharyngeal cancer, nasopharyngeal disease, nasopharyngeal neoplasm, nasopharynx, TB-PCR, tuberculosis

## Abstract

**Rationale:**

Nasopharyngeal tuberculosis (TB), a rare form of tuberculosis outside the lungs, can affect any organ or tissue in the body. It is difficult to diagnose because of nonspecific symptoms, often leading to delayed confirmation after the initial patient visit. Clinical manifestations such as cervical lymphadenopathy and irregular mucosal surfaces can be challenging to distinguish from nasopharyngeal cancer or malignant lymphoma.

**Patient concerns:**

In this case report, we present a patient initially suspected of having a malignant disease based on abnormal nasopharyngeal imaging findings.

**Diagnoses:**

Further examination revealed chronic granulomatous inflammation, and subsequent tuberculosis polymerase chain reaction (TB-PCR) confirmed the diagnosis of tuberculosis.

**Interventions:**

The patient is currently receiving anti-TB treatment with a 4-drug regimen, which has shown a good response with continuous reduction in lesion size.

**Outcomes:**

After anti-TB treatment, the lesion size gradually decreased and continued to decrease, showing a significant response.

**Lessons:**

Awareness and precise evaluation are key to avoiding misdiagnosis, particularly when confronted with diverse clinical presentations. Extrapulmonary tuberculosis, although relatively rare, presents unique diagnostic challenges. Nasopharyngeal tuberculosis, in particular, lacks a definitive diagnostic method, often necessitating a combination of clinical suspicion, imaging studies, microbiological tests, and histopathological examination for confirmation. The absence of specific symptoms and the variability in presentation further compound the diagnostic dilemma. Given the potential consequences of misdiagnosis, further exploration and discussion on this issue are warranted. Enhanced awareness among healthcare providers, coupled with advancements in diagnostic modalities, are essential in ensuring timely and accurate differentiation between nasopharyngeal malignancies and tuberculosis, thereby facilitating appropriate management and improving patient outcomes.

## 1. Introduction

Tuberculosis is a chronic granulomatous infectious disorder caused by Mycobacterium tuberculosis, primarily due to the inhalation of Mycobacterium-impregnated airborne droplets. And It is a significant global health issue that has been a leading cause of morbidity and mortality worldwide for decades.^[1]^ Before the COVID-19 pandemic, tuberculosis was the leading cause of death due to a single infectious agent.^[2]^ While tuberculosis can affect any organ or tissue in the body, nasopharyngeal tuberculosis is a rare type of extrapulmonary tuberculosis that may be primary or secondary to tuberculosis outside the lungs.^[3]^ Nasopharyngeal tuberculosis is uncommon, and its symptoms are nonspecific, making it difficult to differentiate it from other diseases.^[4]^ This often leads to a significant delay in diagnosis, with considerable time between the initial patient visit and confirmed diagnosis. Clinically, cervical lymphadenopathy is the most common symptom of nasopharyngeal cancer or malignant lymphoma, and irregular surfaces or masses in the nasopharyngeal mucosa are often difficult to differentiate from tuberculosis on imaging findings.^[4,5]^

In this case report, we present a patient who was initially suspected of having malignant disease due to abnormal nasopharyngeal imaging findings. However, the patient was eventually diagnosed with chronic granulomatous inflammation on tissue examination and subsequently confirmed to be positive for tuberculosis polymerase chain reaction (TB-PCR), making differential diagnosis difficult. Since extrapulmonary tuberculosis is a rare condition and there is no clear diagnostic method, further discussion of this issue is warranted.

## 2. Case report

**Figure 1.**
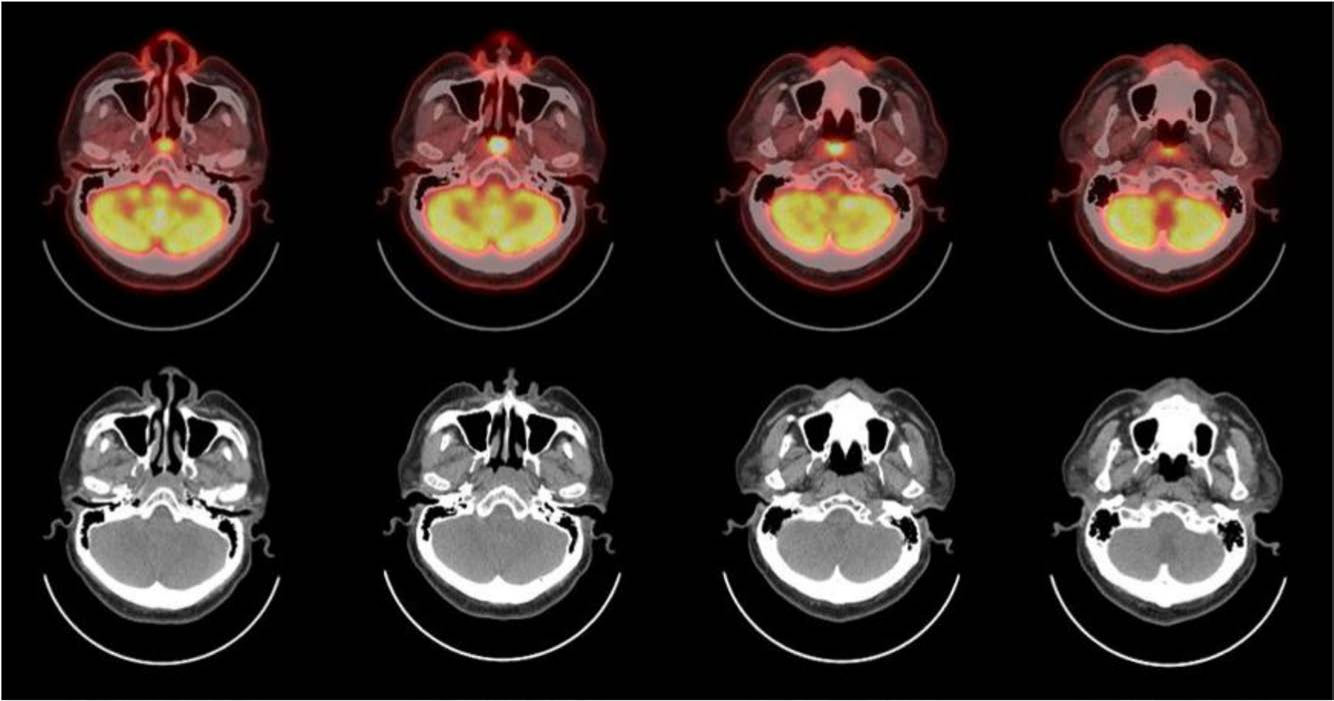
Axial ^18^F-FDG PET and PET/CT images in a 64-year-old man with nasopharyngeal tuberculosis. Pretreatment images show an abnormal ^18^F-FDG uptake on initial PET/CT over the nasopharynx, posterior wall. Malignant lesions could not be excluded and tissue confirmation was recommended. ^18^F-FDG = ^18^F-fluorodeoxyglucose, PET/CT = positron emission tomography/computed tomography.

**Figure 2.**
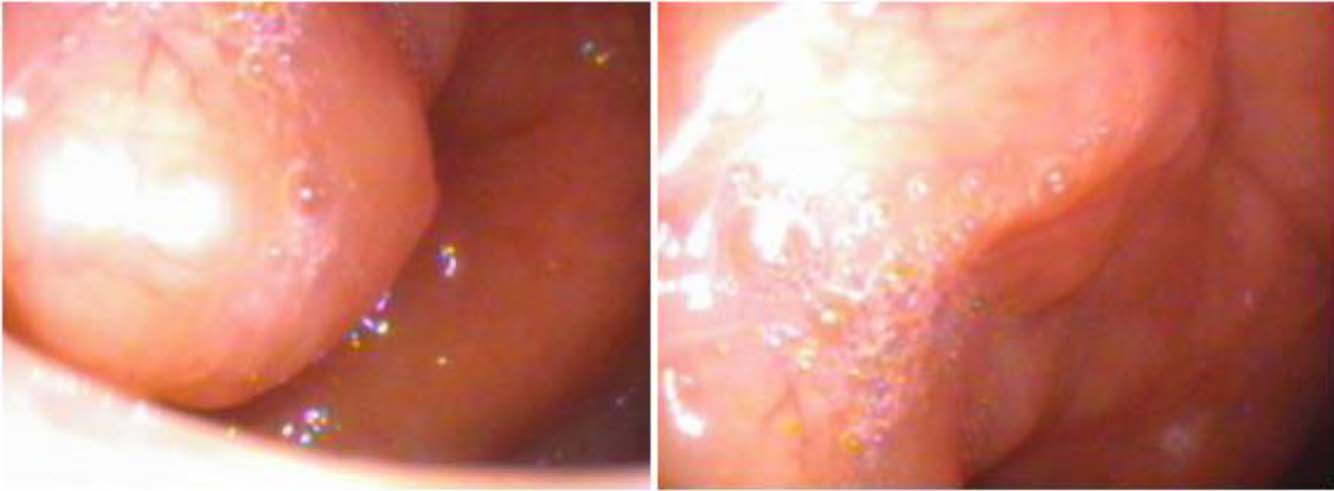
Endoscopic findings of the nasopharyngeal mass. Nasopharynx was filled with round and smooth mass.

**Figure 3.**
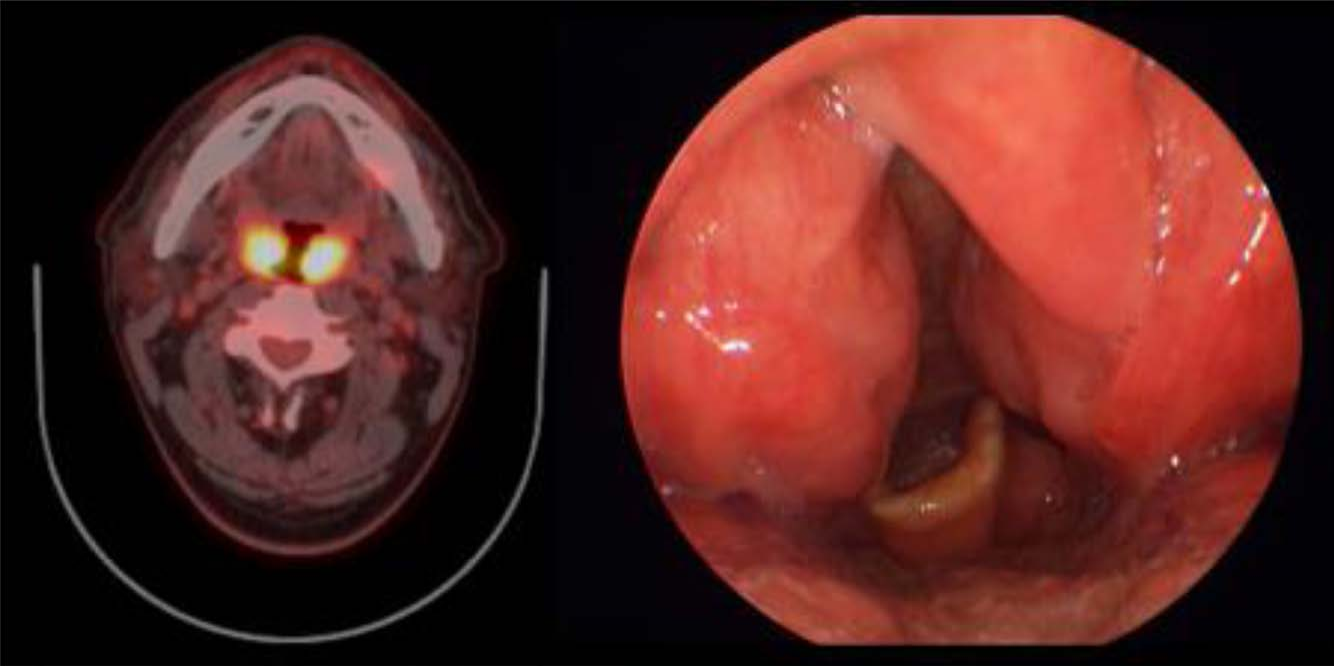
Axial ^18^F-FDG PET and PET/CT images with an abnormal ^18^F-FDG uptake on bilateral tonsils. endoscopic findings showed palatine tonsilar hypertrophy. ^18^F-FDG = ^18^F-fluorodeoxyglucose, PET/CT = positron emission tomography/computed tomography.

**Figure 4.**
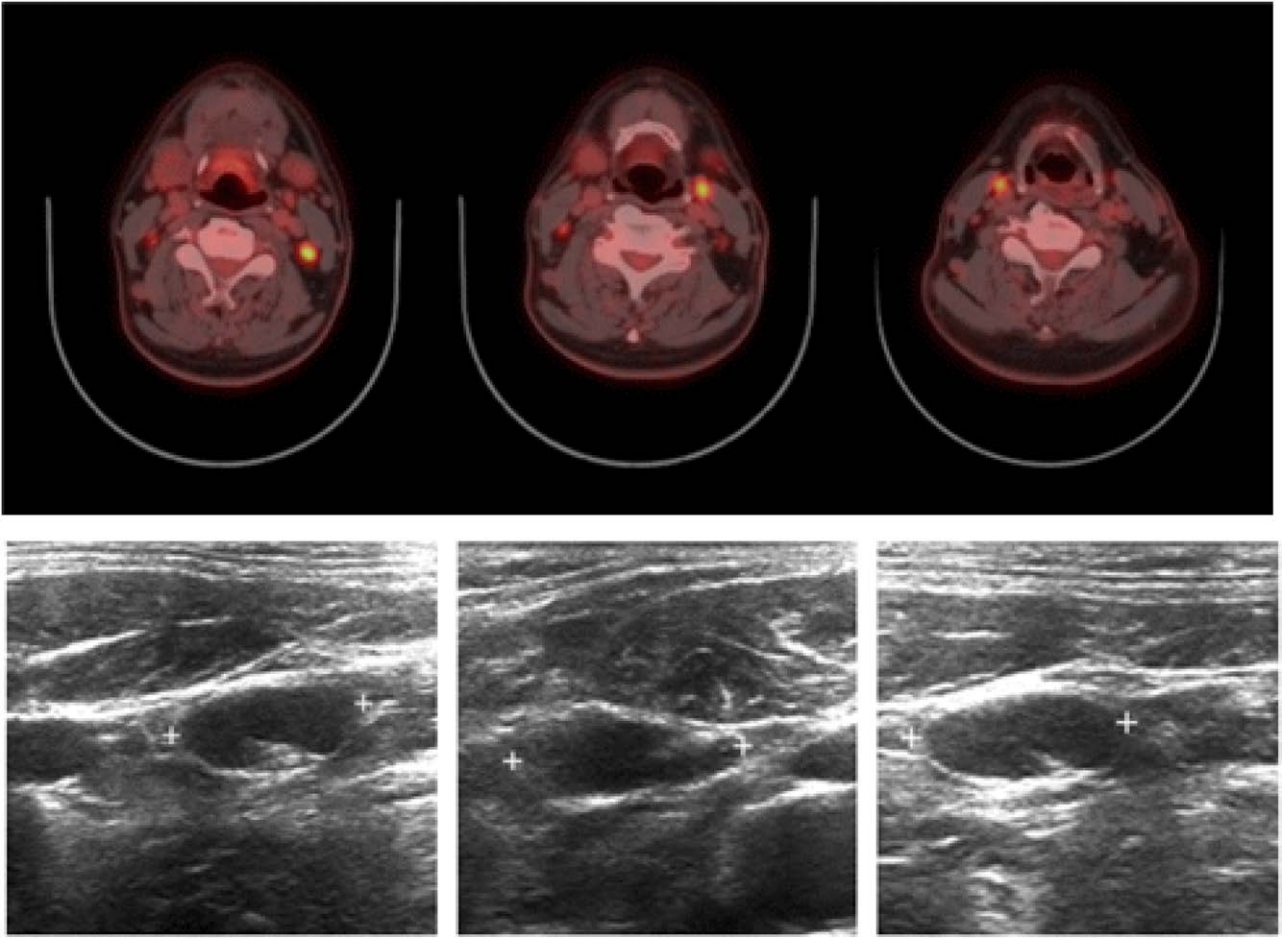
Axial ^18^F-FDG PET and PET/CT images with an abnormal ^18^F-FDG uptake on bilateral neck lymph nodes. Ultrasound shows that the involved nodes are hypoechoic with echogenic thin layers. ^18^F-FDG = ^18^F-fluorodeoxyglucose, PET/CT = positron emission tomography/computed tomography.

**Figure 5.**
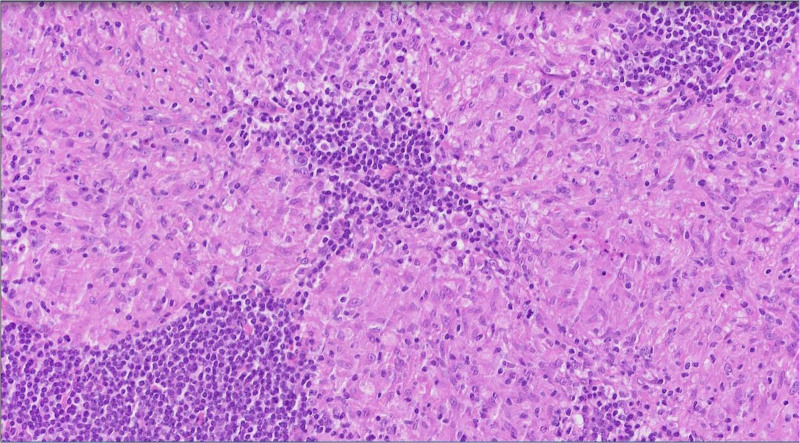
Pathologic findings of nasopharyngeal tuberculosis (H&E stain, X 20).

**Figure 6.**
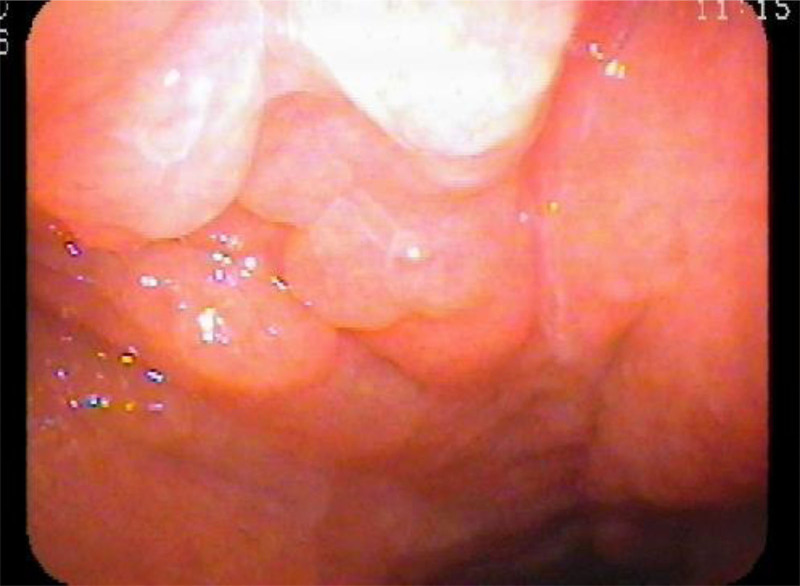
Endoscopic findings of the reduced size of nasopharyngeal mass lesion.

A 64-year-old male patient underwent chest computed tomography (CT) as part of a health checkup, which revealed a nodule in the right upper lobe of the lung. The patient was referred to the pulmonology department for further evaluation and underwent CT-guided percutaneous needle biopsy, bronchoscopy, and positron emission tomography-computed tomography. Percutaneous needle biopsy revealed a few atypical necrotic squamous cells, and bronchoscopy did not reveal any specific findings. Additionally, acid fast bacilli (AFB) staining and culture, real-time polymerase chain reaction, and cytology examination performed on bronchoalveolar lavage did not reveal any abnormal findings. However, positron emission tomography/computed tomography (PETCT) revealed increased uptake in the posterior wall of the nasopharynx, bilateral tonsils, and multiple cervical lymph nodes, which could not exclude the possibility of malignancy. Therefore, the patient was referred to the otolaryngology department for further evaluation (Fig. 1). During the outpatient visit, laryngoscopy and flexible nasopharyngoscopy revealed bilateral hypertrophy of the palatine tonsils and a mass-like lesion in the nasopharynx (Figs. 2 and 3). Needle aspiration biopsy of the left cervical lymph node showed benign cytologic findings (Fig. 4). However, histopathological examination of the nasopharyngeal mass revealed a chronic granulomatous inflammation. (Fig. 5) Subsequently, immunohistochemical staining and nested polymerase TB-PCR were performed, and the nested TB-PCR results were positive. The patient was diagnosed with extrapulmonary tuberculosis (TB) and is currently receiving anti-TB treatment with a 2-drug regemen (rifampicin, pyridoxine) for 3 months initally and 4-drug regimen (rifampicin, pyridoxine ethambutol, pyrazinamide) for 9 months additionally, which has shown a good response with continuous reduction in lesion size (Fig. 6). The patient is currently being followed-up with nasopharyngeal endoscopic exam monthly and there is no evidence of recurrence. Informed consent was obtained from the patient for publication of the case.

## 3. Discussion

Recently, the incidence of extrapulmonary TB has increased. However, in the case of TB involving the nasopharynx, there is no clear diagnostic method described in the literature, even in TB endemic regions.^[3,6]^ Nasopharyngeal TB is difficult to diagnose clinically because of its anatomical structure and nonspecific clinical symptoms, which may lead to the misdiagnosis of malignant tumors.^[4,7]^ Differential diagnoses include malignancy such as squamous cell carcinoma and lymphoma, fungal infections such as aspergillosis and mucormycosis, granulomatous inflammation such as sarcoidosis, leprosy, syphilis and tuberculosis, and autoimmune diseases.^[8,9]^ Primary infection of nasopharyngeal TB occurs through direct spread via nasal ventilation, and secondary infection is mainly transmitted hematogenously or lymphatically from adjacent pulmonary regions.^[10]^

Clinically, nasopharyngeal TB may show irregular mucosal hypertrophy, nodular masses, granulomatous masses, mucosal swelling, and congestion on endoscopic examination; however, these are not characteristic, and typical findings may not be observed on imaging studies such as CT or magnetic resonance imaging, making it difficult to diagnose the disease at an early stage.^[5,11,12]^ Especially, cervical lymphadenopathy, the most common symptom of nasopharyngeal TB, can also appear similarly in nasopharyngeal cancer; therefore, histopathological diagnosis is important to avoid misdiagnosis.^[13,14]^

The diagnosis of nasopharyngeal TB is based on histopathological and microbiological findings of the biopsy tissue.^[8,15]^ Histopathological examination typically shows granulomatous inflammation, accompanied by giant cells and caseous necrosis.^[16]^ However, confusion may arise in the diagnosis of nasopharyngeal cancer with a lesion showing a granulomatous reaction in the tissue adjacent to the tumor. Ziehl–Neelsen staining can detect AFB, but as it takes several weeks to culture them, nucleic acid amplification tests such as polymerase chain reaction (PCR) have been used as a standard method for identifying TB bacteria.^[13,16,17]^

The treatment of extrapulmonary TB is the same as that of pulmonary TB, except in cases in which a specific microorganism is confirmed or there is no resistance to the initial drugs. If anti-TB therapy is administered, nasopharyngeal TB has a good prognosis, and the treatment period is periodically evaluated to determine whether the treatment duration should be extended.^[6,18]^

In some other cases, the histological examination is consistent with TB, but the PCR test results are negative. In cases where the diagnosis is difficult, attempting anti-TB therapy and observing the lesion’s improvement can lead to a diagnosis. Therefore, histological examination and molecular biological techniques such as TB-PCR should be performed complementarily for diagnosis. Given the rare occurrence of nasopharyngeal TB, careful attention is required in clinical practice.

The statistics suggesting that the COVID-19 pandemic disrupted decades of global progress in decreasing TB mortality, and the total number of TB-related deaths in 2020 has reverted to the same level observed in 2017.^[19]^

## 4. Conclusion

Due to the variability of clinical manifestations, awareness of this disease and precise evaluation of the patient were the main considerations to avoid misdiagnosis. Differentiating between malignant diseases involving the nasopharynx and nasopharyngeal tuberculosis is important because the clinical presentations, including cervical lymph node enlargement and lesions in the nasopharynx, are similar. Tissue diagnosis should be considered in all cases, and typical histopathology with caseating granulomatous inflammation must be identified. In highly suspicious cases, PCR analysis, bacterial culture, and AFB staining should be performed for the diagnosis.

## Acknowledgments

This research was supported by Basic Science Research Program through the National Research Foundation of Korea (NRF) funded by the Ministry of Education (2021R1F1A1052136).

## Author contributions

**Conceptualization:** Gil Joon Lee.

**Data curation:** Da Hyun Chung.

**Investigation:** Da Hyun Chung.

**Supervision:** Gil Joon Lee.

**Validation:** Da Hyun Chung.

**Visualization:** Da Hyun Chung, Gil Joon Lee.

**Writing – original draft:** Da Hyun Chung.

**Writing – review & editing:** Da Hyun Chung, Gil Joon Lee.
